# Evaluation of Smartphone Camera Positioning on Artificial Intelligence Pose Estimation Accuracy for Exercise Detection: Observational Study

**DOI:** 10.2196/82412

**Published:** 2026-03-05

**Authors:** Eduarda Oliosi, Soraia Ferreira, Ana Paula Giordano, Guilherme Viveiros, José Parraca, Paulo Pereira, Federico Guede-Fernández, Salomé Azevedo

**Affiliations:** 1 Value for Health CoLAB, 15 Fontes Pereira de Melo Ave, 2nd Fl, Right, Lisbon, 1050‑115, Portugal, 351 937091767; 2 Laboratory for Instrumentation, Biomedical Engineering and Radiation Physics (LIBPhys‑UNL), Physics Department, Nova School of Science and Technology, Nova University of Lisbon, Caparica, Portugal; 3 Research Centre for Physical Activity, Health and Leisure (CIAFEL), Faculty of Sport, University of Porto, Porto, Portugal; 4 Departamento de Desporto e Saúde, Escola de Saúde e Desenvolvimento Humano, Universidade de Évora, Évora, Portugal; 5 Comprehensive Health Research Centre (CHRC), Escola de Saúde e Desenvolvimento Humano, Departamento de Ciências Médicas, Universidade de Évora, Évora, Portugal; 6 Research Unit in Business and Economics, Católica Lisbon School of Business and Economics (CUBE), Catholic University of Portugal, Lisbon, Portugal; 7 Department of Machine Learning, Dotmoovs, Braga, Portugal; 8 Department of Engineering and Management, Instituto Superior Técnico (CEG‑IST), University of Lisbon, Lisbon, Portugal; 9 Comprehensive Health Research Center (CHRC), Nova Medical School, Nova University of Lisbon, Lisbon, Portugal

**Keywords:** computer vision, digital health, human pose estimation, human activity recognition, mobile health, mhealth, mobile apps, physical activity

## Abstract

**Background:**

Artificial intelligence (AI)–driven pose estimation (PE) offers a scalable and cost-effective solution to track exercises in mobile health apps. However, occlusion, influenced by camera angle and distance, can reduce detection accuracy and repetition counting precision. The influence of smartphone positioning on these performance metrics remains underexplored in controlled studies.

**Objective:**

The study aimed to examine how smartphone camera angle (front, side, and diagonal) and distance (90 cm, 180 cm, 200 cm, and 360 cm) affect detection performance and repetition counting accuracy during push-ups and squats using AI-based PE.

**Methods:**

In this cross-sectional, within-subject study, 44 healthy university students (9 [20.5%] female participants; mean age 20.3 y, SD 0.4 y; mean BMI 23.2, SD 0.6 kg/m²) were assigned to perform either squats or push-ups. Each participant completed their assigned exercise across 12 predefined smartphone camera configurations, yielding approximately 264 squat trials (n=22) and 264 push-up trials (n=22). Each trial consisted of an average of 5 repetitions, totaling approximately 1320 repetitions per exercise. PE performance was assessed using binary classification accuracy, detection rate, and mean absolute error (MAE) for repetition counting. Generalized linear mixed-effects models evaluated classification odds, linear mixed-effects models analyzed MAE, and Tukey-adjusted post hoc tests followed significant effects.

**Results:**

The mean detection rate was 61.1% (SD 48.8%) for push-ups and 61.5% (SD 48.7%) for squats, with MAEs of 1.08 (SD 1.78) and 1.11 (SD 1.82) repetitions, respectively. Push-ups were most accurately detected from diagonal views at 90 to 180 cm (up to 85.7% detection; MAE=0.28) and least accurately from the front at 360 cm (20%; MAE=2.70). Squats performed best from a diagonal view at 200 cm (95.5%; MAE=0.05) and worst from the side at 90 cm (0%; MAE=5). Generalized linear mixed models showed that for push-ups, the front 90 cm and diagonal 360 cm views significantly reduced classification odds compared to the side 90 cm view (*P*=.03 and *P*=.04, respectively), whereas for squats, diagonal and front views significantly outperformed side views across all distances (*P*<.001). Post hoc tests confirmed that for push-ups, diagonal close or mid-range views had significantly lower MAEs than far front views, and for squats, diagonal and front views at 180 to 200 cm achieved the highest accuracy and lowest MAEs (*P*<.05).

**Conclusions:**

AI-based PE effectiveness for exercise tracking is significantly affected by smartphone positioning. Diagonal and frontal views at mid-range distances (180‐200 cm) provided the highest detection accuracy and counting precision. These findings offer actionable guidance for developers, clinicians, coaches, and users optimizing mobile health exercise monitoring.

## Introduction

Physical inactivity is a major global public health concern, contributing to higher rates of chronic diseases, reduced quality of life, and increased health care costs [1-3]. While the health benefits of regular physical activity are well documented, ranging from improved cardiovascular fitness and muscle strength to enhanced mental well-being, participation levels remain insufficient worldwide [4]. Current estimates indicate that over 31% of adults and 81% of adolescents fail to meet the World Health Organization’s recommended physical activity guidelines [5]. The consequences of physical inactivity are particularly severe for populations managing chronic or neurological conditions, for whom movement-based interventions can serve as both preventive and therapeutic measures [3,6,7].

In response to the demand for scalable and accessible physical activity tools, mobile health (mHealth) apps have emerged as a cost-effective means of promoting exercise participation [3]. A notable innovation in this field is pose estimation (PE), or markerless motion capture, which uses computer vision to detect joint positions from standard video footage without the need for specialized equipment [8]. Open-source PE models, such as OpenPose (Carnegie Mellon University), BlazePose (Google), and MoveNet (Google), facilitate real-time PE via smartphones or webcams. This enables the provision of remote, personalized feedback [9-13]. In biomedical contexts, PE can identify movement impairment patterns and support rehabilitation or neurological diagnoses [8]. It is also widely applied in fitness, telerehabilitation, occupational health, and sports to track joint angles, velocity, form, and repetitions [14-18]. Validation studies have demonstrated strong agreement with the gold standard motion capture [19-21], and recent studies integrating deep learning have reported repetition-counting accuracies above 90% [22-25].

Despite recent advances, most PE systems have only been evaluated in controlled laboratory environments. However, real-world mHealth apps occur in uncontrolled settings where factors such as handheld devices held at different distances and angles, and partial obstructions (eg, from pets, furniture, or body position) can significantly degrade performance [10,12,26]. These challenges are particularly pronounced for dynamic, whole-body movements such as squats and push-ups, where tracking accuracy can vary depending on body position and elevation. Although a few commercial solutions have begun to experiment with camera-based self-assessment (eg, Halo Movement [Amazon] [27] and Kaia Health GmbH [28]), there is still a lack of empirical studies on how smartphone positioning affects the reliability of PE in practical settings [10,14,27]. Improving accuracy in these contexts will likely require advances not only in 2D keypoint detection but also in our understanding of optimal camera placement and how to transform 2D keypoint data into accurate spatial representations.

This study systematically evaluates the impact of smartphone camera angle and distance on the performance of artificial intelligence (AI)–based PE when counting 2 foundational bodyweight exercises: squats and push-ups. These exercises are commonly used for fitness and rehabilitation tracking. They involve multijoint movements and present common occlusion challenges. Repetition counts are obtained from 2D PE across 12 camera configurations (3 angles × 4 distances) and benchmarked against expert-labeled ground truth. The aim is to examine how smartphone recording position, specifically camera angle and distance, affects detection performance and repetition counting accuracy, and to inform best practices for using mHealth systems in real-world environments. This work is part of the Blockchain.PT initiative (Project No. 51), which supports sustainable blockchain-based digital innovation in the health and sports sectors.

## Methods

### Study Design

This cross-sectional, within-subject study was designed to assess the influence of smartphone camera configurations, specifically angle and distance, on the detection of PE during squats and push-ups. These exercises were selected due to their complex multijoint biomechanics and typical self-occlusions (eg, torso obscuring limbs in push-ups).

### Setting

All data were collected in a controlled laboratory environment at the School of Health and Human Development, University of Évora, Portugal, in May 2025. Environmental conditions, including lighting and temperature, were standardized across trials. Smartphone placement was controlled at predefined angles and distances to ensure reproducible video capture. The laboratory provided enough space for participants to safely perform push-ups and squats.

### Participants

Participants were recruited from undergraduate courses at the University of Évora in Portugal through a targeted study announcement that specified the eligibility criteria, study procedures, and recommended attire for physical exercise during classes. Eligible volunteers were identified through convenience sampling with eligibility screening.

A total of 44 healthy university students participated and self-reported their status. Inclusion criteria were: (1) aged 18 years or over; (2) able to safely perform squats and push-ups; (3) not having used sedative or balance-impairing medications within the last 24 hours; and (4) absence of neurological or musculoskeletal conditions that could impair movement.

### Ethical Considerations

The study protocol was reviewed and approved by the Ethics Committee of the University of Évora (GD/27378/2024), in accordance with institutional and international standards for human participant research. All procedures complied with the Declaration of Helsinki and the General Data Protection Regulation. Written informed consent was obtained from all participants prior to enrollment. Participants were informed about the study objectives, procedures, and potential risks and were explicitly advised of their right to withdraw at any time without penalty. To ensure privacy and confidentiality, all collected data were anonymized and coded to prevent participant identification. Data were securely stored on password-protected institutional servers accessible only to the research team. No monetary or material compensation was provided for participation in this study.

### Exercise Protocol and Dataset

Participants were first briefed on the study protocol and completed a demographic questionnaire to record baseline characteristics. For the squat task, participants stood with feet shoulder-width apart and arms extended forward, performing repetitions by lowering their hips until their thighs were parallel to the floor. Male participants performed push-ups in a standard plank position, while female participants were allowed a modified knee-supported variation. All exercise procedures adhered to the 11th edition of the *American College of Sports Medicine* guidelines (2021) [29], which recommend these variations to accommodate differences in strength and fitness levels. To reduce fatigue-related performance decline and ensure high-quality movement data, participants were assigned to perform either squats or push-ups based on personal preference. Of the 44 participants, 22 (16 male participants and 6 female participants) completed the squat protocol, and 22 (19 male participants and 3 female participants) completed the push-up protocol. Each participant performed their assigned exercise across all 12 predefined camera configurations, yielding 264 squat trials (22×12) and 264 push-up trials (22×12). Each trial consisted of a continuous movement sequence, averaging approximately 5 repetitions per video [16], totaling approximately 1320 repetitions per exercise type (264 trials × ~5 reps).

### Experimental Setup

To capture a representative sample of commonly used consumer devices, exercise sessions were recorded using three smartphone models, iPhone 11 (Apple Inc.), iPhone 13 (Apple Inc.), and Samsung Galaxy A52 (Samsung Electronics), each capturing video at 1080 p resolution and 30 frames per second. Smartphones were positioned horizontally on the floor at 3 fixed angles relative to the participant’s body: frontal (0°), diagonal (45°), and lateral (90°). Recordings were conducted at 4 distances, including 180 cm based on prior literature [30], and 3 additional distances (90 cm, 200 cm, and 360 cm) defined by the research team to test a wider range of realistic recording conditions. This configuration resulted in 12 distinct camera setups (3 angles × 4 distances). All smartphone positions were marked and standardized to ensure consistency across trials.

This experimental setup is contextualized within existing research by Table 1, which summarizes previous studies on push-up and squat detection, detailing camera perspectives, distances, PE methods, and reported performance metrics. The comparison shows that most prior studies rely on limited viewpoints and fixed distances, restricting generalizability. In contrast, our multiangle, multidistance dataset addresses these constraints, providing a more diverse and representative resource for evaluating PE performance.

**Table 1.** Summary of state-of-the-art push-up and squat pose estimation datasets and methods.

Reference	Exercise	Camera perspectives	Camera distances	PE^n^ method	Instrument	Key evaluation metrics	Dataset
Park et al [31]	Push-up	Frontal, side	Full body visible, N/R^b^ value	OpenPose	2 cameras	ACC^c^=90%	Custom dataset of n=12
Youssef et al [32]	Squat	Frontal, side	N/R	BlazePose	Mobile devices + inertial sensors	ACC=94%	EJUST-SQUAT-21^o^, single individual, MM-Fit^e^ datasets
Hande et al [33]	Squat and others	Frontal, side	Full body visible, N/R value	OpenPose, MobileNet (Google), InceptionV3 (Google)	Single camera	ACC =~98% (MobileNet),~96% (InceptionV3)	Penn Action Dataset
Chae et al [34]	Squat	Frontal, diagonal	250 cm Kinect (Microsoft), 380 cm webcam	OpenPose, Temporal Conv1D^f^, BiLSTM^g^	Kinect + webcam	ACC=85%	Custom dataset of n=52
Chariar et al [35]	Squat	Frontal	Distance N/R; 120 cm height	MediaPipe (Google), Bi-GRU^h^	2 depth cameras	ACC=94%	Custom dataset of n~50
Zhang et al [36]	Push-up	Frontal, side	N/R	MoveNet; angle-heuristic; Optical flow	N/R	Average *F*_1_-score: angle-heuristic=0.85 (side > front); pose classification=0.94 (side > front); optical flow=0.79 (front > side)	Kaggle “Push-up Exercise” dataset
Japhne et al [24]	Push-up, squat, and others	N/R, full body visible	200 cm	OpenPose, LSTM^i^	Mobile devices	Push-up: ACC=~99%; Squat: ACC=~99%	Custom dataset of n=3
Mercadal-Baudart et al [21]	Squat and others	Frontal; multiangle validation	~300 cm radius; 150 cm height	Detectron2 (Meta Platforms, Inc), Strided Transformer	Mobile devices	RMSE^j^ of joint angles versus VICON^k^ (Vicon Motion Systems): <10° for most joints (shin, knee, hip, trunk, and spine), <15° for shoulder and ASIS^l^ (notably front squats)	Custom dataset of n=8‐12

a PE: pose estimation.

b N/R: not reported.

c ACC: accuracy.

d EJUST-SQUAT-21: Egypt-Japan University squat dataset 2021.

e MM-Fit: multimodal fitness dataset.

f Conv1D: one-dimensional convolutional neural network.

g BiLSTM: bidirectional long short-term memory.

h Bi-GRU: bidirectional gated recurrent unit.

i LSTM: long short-term memory.

j RMSE: root mean square error.

k VICON: Vicon motion capture system.

l ASIS: anterior superior iliac spine.

### Pose Estimation and Processing

Video data were processed using a multistage PE and repetition detection pipeline designed for real-world deployment on the Dotmoovs mobile platform [37]. The pipeline was optimized for low-latency, on-device inference while maintaining sufficient accuracy for exercise recognition.

#### Model Selection

A lightweight 2D PE model was prioritized to ensure real-time performance on consumer smartphones and without requiring external sensors. Among candidate architectures (eg, PoseNet [Google], BlazePose, and MoveNet), MoveNet was selected due to its computational efficiency, suitability for edge inference, and enhanced generalization to fitness-related movements. MoveNet was trained on the COCO (Common Objects in Context) dataset (Microsoft) and Google’s internal Active dataset, which includes annotated yoga, fitness, and dance poses exhibiting substantial motion variability [38-40]. For cloud-based processing, EvoPose2D (Huawei Technologies Co, Ltd) and ViTPose (Microsoft Research Asia) were implemented to achieve higher precision at the cost of increased computational load. This hybrid configuration balances latency, accuracy, and hardware constraints, supporting both on-device responsiveness and scalable cloud inference [39]. The models were trained on approximately 1 million samples, reserving 10,000 samples each for validation and testing, ensuring robustness and generalizability across diverse movement types.

#### Training Datasets and Domain Adaptation

To improve robustness for fitness-specific postures, the training pipeline incorporated multiple publicly available datasets: SMART (Sports Motion and Recognition Tasks) [41], LSP (Leeds Sports Pose) Extended [42], Penn Action [43], and MPII Human Pose [44]. Despite these datasets, complex movements, particularly floor-based exercises (eg, push-ups), remained challenging for accurate PE. This motivated the creation of DotPose, a custom internal dataset designed to complement existing datasets and improve the detection of occluded limbs and challenging postures. The combination of DotPose with public datasets further mitigates potential biases related to body types, cultural differences, exercise contexts, and environmental scenarios, thereby enhancing generalizability across diverse users and real-world conditions.

#### Pipeline Implementation

The PE outputs (17 keypoints per frame) were processed through a proprietary deep learning module performing two primary tasks:

1. State classification: A lightweight neural network classifies each frame into discrete movement states (eg, squat-down, squat-up, or other).
2. Repetition counting: A Markov-chain-based algorithm tracks temporal transitions between states, triggering a repetition event when a predefined sequence (eg, up-down-up) is detected.

This architecture ensures temporal stability and robustness against intermittent keypoint noise. The pipeline was benchmarked for real-time inference, with MoveNet demonstrating subsecond latency, suitable for interactive mobile feedback. Finally, all pose outputs were exported in comma-separated values format to enable statistical analysis of accuracy and classification metrics across varying camera angles and distances.

### Manual Annotation

All video samples were independently annotated by 2 trained raters (an exercise physiologist and a physical therapist), who followed a predefined schema based on standardized exercise movement criteria. Each annotator labeled the exercise type and manually counted the number of valid repetitions per trial. No discrepancies were observed between raters. These annotations were then used to evaluate the accuracy of detection and repetition counting.

### Statistical Analysis

All analyses were conducted in RStudio (version 4.5.0; R Foundation for Statistical Computing) using established packages including lme4, lmerTest, emmeans, and performance. Performance metrics were reported in alignment with prior benchmark studies in human activity recognition and AI-based motion analysis [26,45]. The primary outcome was a binary accuracy indicator, coded as 1 when the system’s predicted repetition count exactly matched the ground truth, and as 0 otherwise. This was modeled using generalized linear mixed-effects models (GLMMs) with a logit link. The secondary outcome was the mean absolute error (MAE), defined as the absolute difference between predicted and ground-truth repetition counts per video, modeled using linear mixed effects models (LMMs) with a Gaussian distribution. For descriptive reporting purposes, we also calculated a detection rate for each video, defined as (prediction or ground truth) x100, representing the percentage of repetitions counted correctly. Both models included camera angle and distance as fixed effects, with participant ID modeled as a random intercept to account for intraindividual variability and repeated measures. When the fixed effects were statistically significant (*P*<.05), Tukey-adjusted post hoc contrasts were conducted to compare condition pairs. Odds ratios (ORs) were computed by exponentiating the log odds from the GLMM to facilitate interpretation.

## Results

### Participant Characteristics

Our dataset comprised 44 healthy university students from the University of Évora, Portugal, including 9 (20.5%) females and 35 (79.5%) males. The participants had a mean age of 20.31 (SD 0.40) years, a mean height of 1.74 (SD 0.09) meters, and a mean body mass of 70 kg (SD 14.72), resulting in a mean BMI of 23.16 kg/m² (SD 0.61).

Based on previous studies assessing AI-based PE for push-up and squat detection [24], we assumed a medium-to-large expected effect size (Cohen *d*=0.65) for power estimation. A post hoc analysis conducted in G*Power 3.1 (Heinrich Heine University Düsseldorf) indicated that the current sample size (n=44) provides approximately 0.74 statistical power to detect this effect at a 2-tailed significance level of *α*=.05, suggesting adequate sensitivity for detecting meaningful differences in PE performance across camera conditions.

### Push-Up Performance

**Figure 1.**
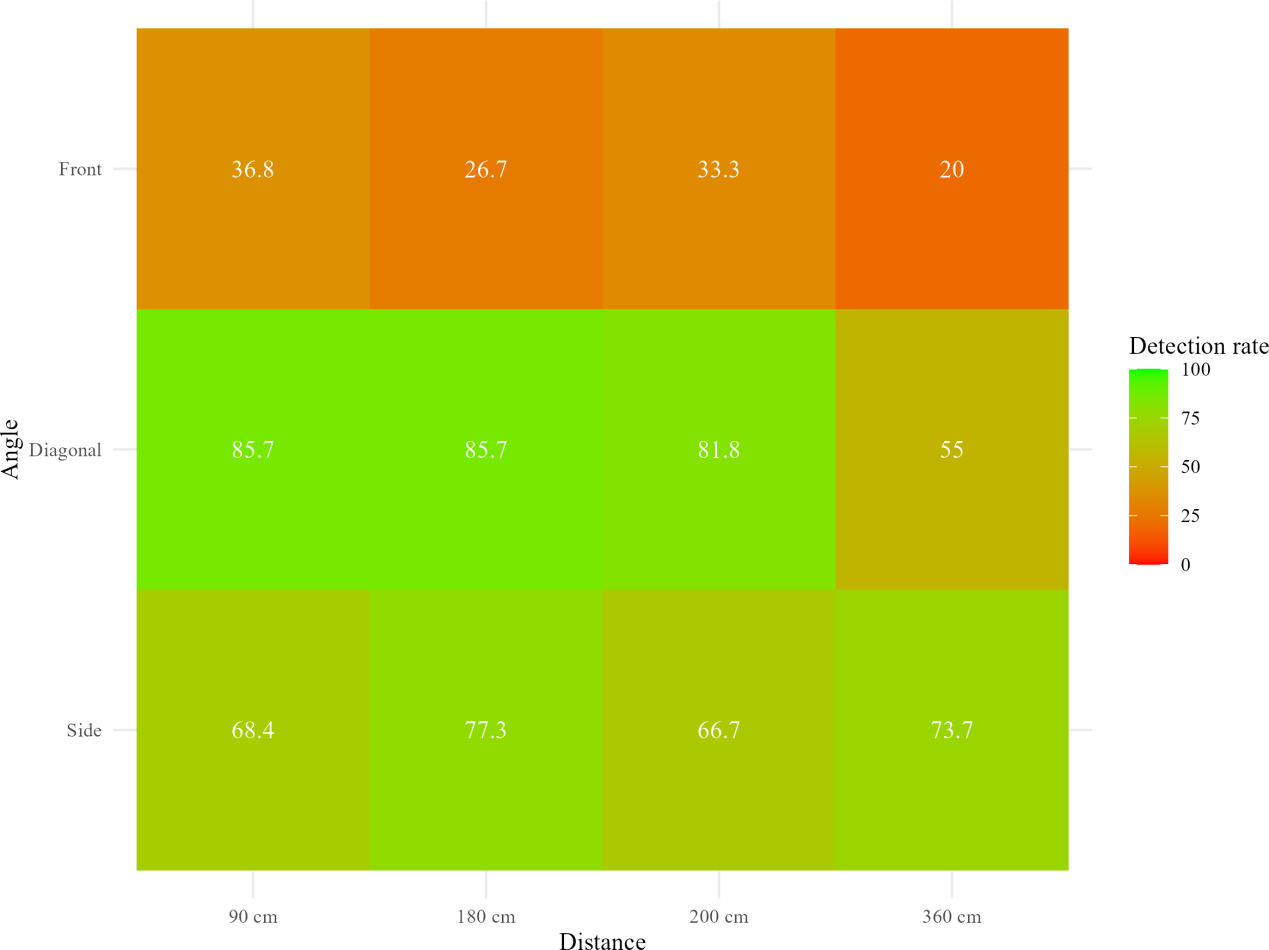
Detection rate of push-ups across distances and angles.

Table 2 shows detection rates and MAEs for push-ups across camera angles and distances. Overall, the mean detection rate was 61.1% (SD 48.8%), with an average MAE of 1.08 (SD 1.78) repetitions. Detection rates peaked for diagonal views at 90 cm and 180 cm (both 85.7%, SD 35.9%), as illustrated in Figure 1. The corresponding MAEs at these distances were low, at 0.29 (SD 0.90) repetitions. In contrast, the front view at 360 cm showed the lowest detection rate of 20% (SD 41.0%) and the highest MAE of 2.70 repetitions (SD 2.05).

**Table 2.** Detection rate and MAE^a^ for push-ups across camera angles and distances.

Camera angle	Distance (cm)	Detection rate (%), mean (SD)	MAE, mean (SD)
Side	90	68.4 (47.8)	0.842 (1.537)
Side	180	77.3 (42.9)	0.409 (0.796)
Side	200	66.7 (48.3)	1.095 (1.972)
Side	360	73.7 (45.2)	0.789 (1.653)
Diagonal	90	85.7 (35.9)	0.286 (0.902)
Diagonal	180	85.7 (35.9)	0.286 (0.902)
Diagonal	200	81.8 (39.5)	0.227 (0.528)
Diagonal	360	55.0 (51)	1.500 (2.482)
Front	90	36.8 (49.6)	1.684 (1.974)
Front	180	26.7 (45.8)	2.067 (2.017)
Front	200	33.3 (48.8)	1.733 (1.981)
Front	360	20 (41)	2.700 (2.055)

a MAE: mean absolute error.

The GLMM (Table S1 in Multimedia Appendix 1) revealed a significant main effect of camera angle on accuracy. Specifically, front views had significantly lower odds of detection (OR=0.17, *P*=.03). However, no significant effects of distance alone were found. Conversely, a statistically significant interaction was found between the diagonal view and the 360 cm distance, which reduced the ability to correctly identify push-ups (*P*=.04).

Post hoc pairwise contrasts were performed to examine differences between the specific camera angles and distances (Table S2 in Multimedia Appendix 1). These contrasts confirmed that diagonal camera views at 90 cm significantly outperformed frontal views at all distances, including 90 cm (estimate=3.41, *P*=.019), 180 cm (estimate=3.67, *P*=.02), 200 cm (estimate=3.52, *P*=.02), and 360 cm (estimate=4.40, *P*<.001). There were also notable differences in the multiple mid-range diagonal and frontal configurations: the diagonal 180 cm view outperformed the frontal 180 cm view (estimate=3.64, *P*=.02), 200 cm (estimate=3.50, *P*=.03), as well as 360 cm (estimate=4.35, *P*=.002).

Likewise, the diagonal 200 cm view surpassed the frontal 360 cm view (estimate=3.96, *P*=.003). Additionally, the side views at 180 cm and 360 cm were significantly better than the frontal 360 cm view (estimate=3.586, *P*=.008, and estimate=3.174, *P*=.03, respectively). These results demonstrate that diagonal and mid-range frontal camera placements substantially increase push-up detection accuracy compared to frontal or side views at extreme distances.

Analyses of push-up repetition MAE using LMM were conducted to evaluate the error comparisons in the respective configurations. Although the main effects of angle and distance were not individually significant beyond the intercept (Table S3 in Multimedia Appendix 1), Tukey’s post hoc tests (Table S4 in Multimedia Appendix 1) revealed meaningful differences between specific camera setups. Diagonal angles at mid-range distances consistently reduced counting errors compared with frontal and side angles at extreme distances. Diagonal views at 90 cm and 180 cm, for example, significantly outperformed frontal views at 180 cm and 360 cm (estimates=−1.686 to −2.395, *P*=.04). Diagonal views at 200 cm were also superior to frontal views at 360 cm (estimate=−2.428, *P*<.001). Side views at extreme distances exhibited higher MAE than frontal views at 360 cm (estimate=−1.800, *P*<.01). Overall, these results suggest that diagonal positioning at mid-range distances (approximately 180R‐200 cm) minimizes counting errors in push-ups.

### Squat Performance

**Figure 2.**
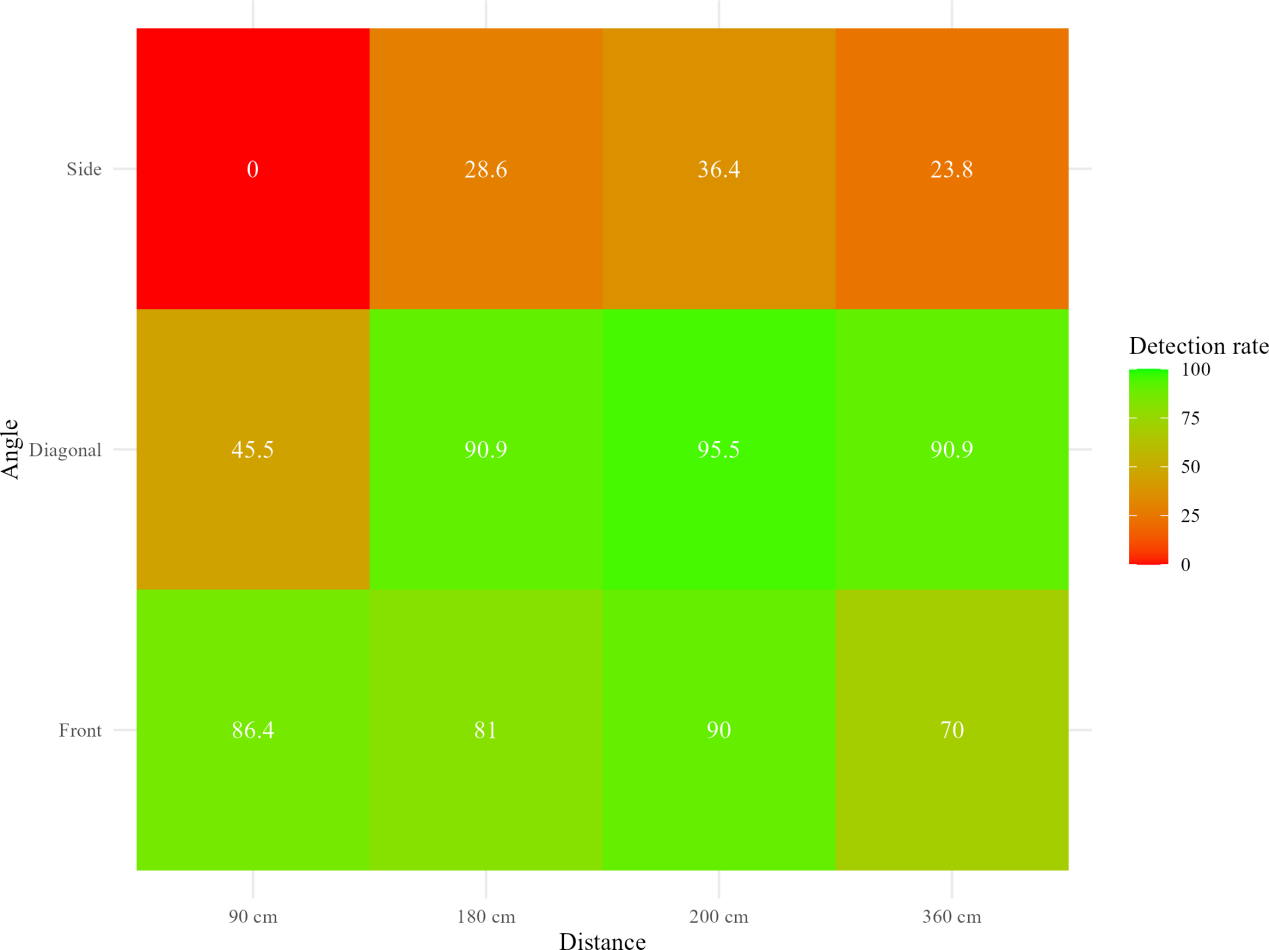
Detection rate of squats across distances and angles.

Table 3 shows detection rates and MAEs for squats across camera angles and distances. Overall, the mean detection rate was 61.5% (SD 48.7%), with an average MAE of 1.11 (SD 1.82) repetitions. Detection rates peaked for the diagonal view at 200 cm (95.5%, SD 21.3%), as illustrated in Figure 2. The corresponding MAE at this distance was minimal, at 0.05 (SD 0.21) repetitions. In contrast, the side view at 90 cm showed the lowest detection rate of 0% (SD 0%) as well as the highest MAE of 5 (SD 0) repetitions.

**Table 3.** Detection rate and MAE^a^ for squats across camera angles and distances.

Camera angle	Distance (cm)	Detection rate (%), mean (SD)	MAE, mean (SD)
Side	90	0 (0)	5 (0)
Side	180	28.6 (46.3)	0.714 (0.463)
Side	200	36.4 (49.2)	0.636 (0.492)
Side	360	23.8 (43.6)	3.095 (2.3)
Diagonal	90	45.5 (51)	1.318 (1.673)
Diagonal	180	90.9 (29.4)	0.091 (0.294)
Diagonal	200	95.5 (21.3)	0.045 (0.213)
Diagonal	360	90.9 (29.4)	0.091 (0.294)
Front	90	86.4 (35.1)	0.318 (0.945)
Front	180	81 (40.2)	0.571 (1.248)
Front	200	90 (30.8)	0.4 (1.273)
Front	360	70 (47)	1.1 (1.889)

a MAE: mean absolute error.

Analyzing the binary accuracy through the GLMM (Table S5 in Multimedia Appendix 1) demonstrated that the diagonal and frontal angles significantly outperformed the side angle at 90 cm (OR=20.43, *P*<.001, and OR=23.01, *P*<.001, respectively). Detection also significantly improved at mid-range distances of 180 cm (OR=4.36, *P*=.002), 200 cm (OR=7.62, *P*<.001), and 360 cm (OR=3.054, *P*=.01) compared to 90 cm. Post hoc pairwise comparisons (Table S6 in Multimedia Appendix 1) revealed that the diagonal view at 180 cm significantly outperformed the front view at 90 cm (estimate=2.810, *P*=.02) and the front view at 180 cm (estimate=3.058, *P*=.009). However, it underperformed compared to the front view at 200 cm (estimate=−3.271, *P*=.01) and the front view at 360 cm (estimate=−4.016, *P*=.02). Side views at 180 cm and 360 cm outperformed the front view at 360 cm in correctly detecting push-ups (estimates=2.907 to 3.239, both *P*=.04). The diagonal view at 200 cm had significantly better detection accuracy than the front view at 360 cm (estimate=3.174, *P*=.03). These findings suggest that diagonal mid-range placements generally perform well, but that frontal views at longer distances (200 and 360 cm) can sometimes outperform the diagonal view at 180 cm for squat detection.

For squats, LMM analysis of MAE (Table S7 in Multimedia Appendix 1) revealed significantly lower errors for diagonal and frontal angles compared with the side (estimates were −3.682 and −4.682, respectively; both *P*<.001). All tested distances (180 cm, 200 cm, and 360 cm) were superior to the 90 cm reference (estimates ranged from −4.364 to −1.910; *P* values<.001). Several significant interactions were observed. For instance, combining a diagonal angle with 180 cm or 200 cm decreased MAE by 3.06 and 3.09 repetitions, respectively (both *P*<.001), which moderated the improvements to the main effects expected from angle and distance independently. The front × 180 cm interaction (estimate=4.547, *P*<.001) and the front × 200 cm interaction (estimate=4.460, *P*<.001) exhibited similar patterns, indicating smaller improvements than the sum of the main effects predicted. In the model, only the diagonal × 360 cm interaction was nonsignificant (*P*=.16). Several significant post hoc pairwise comparisons were identified (Table S8 in Multimedia Appendix 1). These confirmed that diagonal and frontal mid-range placements consistently minimized error. Diagonal at 200 cm produced the largest reduction (estimate=−4.96 repetitions, *P*<.001), similar to diagonal at 180 cm (estimate=−4.91 repetitions, *P*<.001). Most configurations substantially reduced MAE compared to the 90 cm side, but the magnitude of improvement depended on the specific combination of angle and distance. This highlights notable interaction effects.

## Discussion

### Main Findings

This study demonstrates that smartphone camera angle and distance critically affect the accuracy of PE models for detecting and counting push-ups and squats. Consistent with our results, camera placements at intermediate distances (180‐200 cm) combined with oblique (diagonal) or frontal views generally yielded the highest detection rates and lowest counting errors. In contrast, very close-range setups (90 cm) and long-distance frontal views (360 cm) often showed reduced performance. For push-ups, diagonal views between 90 and 200 cm outperformed frontal angles, with the diagonal view at 200 cm position achieving the lowest MAE. For squats, diagonal and frontal views at 180 to 200 cm significantly outperformed side views, with diagonal 180 cm, diagonal 200 cm, and front 200 cm producing the smallest MAEs and the largest error reductions in post hoc tests. Although front 360 cm occasionally approached the accuracy of diagonal 180 cm, most mid-range configurations substantially outperformed both close-range and long-distance side views. These findings provide direct evidence that mid-range diagonal and frontal camera configurations optimize PE performance, informing best practices for smartphone-based exercise monitoring.

### Comparison to Prior Work

Unlike prior lab-based, multicamera studies [11,31], our study provides practical, configuration-specific guidance for monocular smartphone setups. This approach enhances ecological validity by closely mimicking typical home or gym environments where single-camera devices are commonly used. While [17] demonstrated that full-body visibility at 300 cm supports accurate gait tracking, we show that diagonal views at 180 to 200 cm consistently optimize visibility and accuracy for dynamic strength exercises. Views at 360 cm, however, yield variable and often low detection rates (push-ups: 20%‐73.7%; squats: 23%‐90.9%), depending on the angle [46], evaluated smartphone-based distance estimation between 100 and 300 cm, the trade-offs between spatial accuracy, and usability. Our findings confirm that mid-range diagonal placements are optimal for PE performance, likely due to differences in exercise posture (horizontal for push-ups vs upright for squats).

Prior studies have evidenced smartphone apps using TensorFlow Lite and COCO-trained models for exercise counting [10,47], but have not comprehensively examined spatial configuration effects. Our results address this gap, complementing [48] who emphasized viewpoint in PE data acquisition [9], advocating for accessible PE tools beyond labs, and [12] who discussed challenges of motion tracking in naturalistic settings. By offering concrete camera setup recommendations, this work contributes actionable insights for mHealth, home fitness, telerehabilitation, sports performance, and clinical decision-making contexts.

### Practical Implications for mHealth

Positioning the smartphone camera diagonally (~45°) at 180 to 200 cm significantly enhances PE accuracy without additional hardware. Mobile apps can integrate augmented reality or setup guides to assist users in achieving optimal device placement. Accurate repetition counting supports load monitoring, fatigue tracking, and muscular endurance assessment in unsupervised environments [29,49,50]. Combining spatial optimization with adaptive feedback and personalized experiences may further improve tracking reliability and user engagement, consistent with evidence from behavior change and human-computer interaction research [51,52]. This integrated approach presents a promising path for scalable, user-friendly mHealth exercise platforms.

### Limitations

This study has several limitations. First, PE performance was evaluated using manual annotations rather than a gold-standard motion capture system, which may introduce variability. Second, the sample comprised healthy young adults in a controlled laboratory environment, limiting generalizability to clinical populations, older adults, or real-world contexts with variable lighting and backgrounds. Third, while only 1 PE pipeline was tested, integrating multiple public datasets and the custom DotPose dataset helped mitigate biases related to scene composition, body type, exercise variety, and environmental scenarios. Furthermore, comparisons with other open-source PE models (eg, OpenPose, HRNet [Microsoft Research Asia], and BlazePose) are limited by variations in datasets, computational demands, and architectural design [12,35,53]. Finally, lightweight 2D models, such as MoveNet, enable near-real-time, on-device inference. These models balance speed, portability, and user engagement with the fine-grained accuracy achievable by heavier, cloud-reliant models [13]. This hybrid mobile-cloud configuration supports the robust evaluation of push-ups and squats across multiple camera setups. Nevertheless, findings may not generalize to other PE architectures, populations, or unstructured environments.

### Future Directions

Future work should explore multimodal pipelines, diverse participant groups, and variable environmental conditions (eg, low-light and high-contrast conditions) to enhance robustness, applicability, and real-world relevance of mobile PE systems. While repetition counting is a fundamental first step, future mHealth systems should also evaluate movement quality. This should encompass compensatory strategies and fatigue-related adaptations [54-56]. Emerging real-time PE technologies that optimize sensor or camera placement, combined with adaptive feedback responsive to user performance, offer opportunities to enhance movement accuracy and user experience in unsupervised settings [28,48,57,58]. Longitudinal studies are needed to assess the integration of spatial setup guidance, personalization, and real-time feedback for improved usability, engagement, and long-term clinical or fitness outcomes.

### Conclusions

Camera angle and distance significantly affect the accuracy of PE systems for exercise detection. For optimal performance, smartphone cameras should be positioned at mid-range distances (180‐200 cm) with diagonal or frontal views. For push-ups, diagonal views are preferred, while for squats, both diagonal and frontal views perform well. Conversely, close-range setups (90 cm) and long-distance frontal views (360 cm) substantially reduce detection and counting accuracy. These findings provide actionable guidance for developing scalable, accurate, and user-friendly mHealth exercise tracking platforms.

## Supplementary material

10.2196/82412 Multimedia Appendix 1 Detailed results of generalized linear mixed-effects models, linear mixed-effects models, and post-hoc pairwise comparisons for push-ups and squats.

## Abbreviations

AI: artificial intelligence
COCO: Common Objects in Context
GLMM: generalized linear mixed model
LMM: linear mixed effects model
LSP: Leeds Sports Pose
MAE: mean absolute error
mHealth: mobile health
OR: odds ratio
PE: pose estimation
SMART: Sports Motion and Recognition Tasks
