# Supplementary material for: Evaluation of Smartphone Camera Positioning on Artificial Intelligence Pose Estimation Accuracy for Exercise Detection: Observational Study
Source: JMIR Mhealth Uhealth. 2026 Mar 5;14:e82412. doi: 10.2196/82412 (PMC12978916; doi:10.2196/82412)
Supplement: Multimedia Appendix 1 [file mhealth-v14-e82412-s001.doc]

## **Multimedia Appendix - Supplementary Tables S1–S8**

Table S1. Generalized linear mixed model for push-ups

| **Factor** | **Estimate** | **OR**^a^ | **SE**^b^ | **z value** | ***P* value** |
| --- | --- | --- | --- | --- | --- |
| Intercept | 0.958 | 2.607 | 0.684 | 1.400 | .161 |
| Factor(Angle)Diagonal | 1.615 | 5.030 | 0.929 | 1.738 | .082 |
| Factor(Angle)Front | -1.792 | 0.166 | 0.836 | -2.143 | .032 |
| Factor(Distance)180cm | 0.803 | 2.232 | 0.837 | 0.960 | .337 |
| Factor(Distance)200cm | -0.044 | 0.956 | 0.801 | -0.056 | .955 |
| Factor(Distance)360cm | 0.390 | 1.478 | 0.841 | 0.464 | .642 |
| Factor(Angle)Diagonal:Factor(Distance)180cm | -0.839 | 0.431 | 1.304 | -0.644 | .519 |
| Factor(Angle)Front:  Factor(Distance)180cm | -1.068 | 0.343 | 1.232 | -0.866 | .386 |
| Factor(Angle)Diagonal:Factor(Distance)200cm | -0.389 | 0.677 | 1.242 | -0.313 | .754 |
| Factor(Angle)Front:  Factor(Distance)200cm | -0.067 | 0.934 | 1.198 | -0.056 | .955 |
| Factor(Angle)Diagonal:Factor(Distance)360cm | -2.541 | 0.078 | 1.240 | -2.049 | .040 |
| Factor(Angle)Front:  Factor(Distance)360cm | -1.380 | 0.251 | 1.223 | -1.129 | .259 |

^a^OR: Odds Ratio calculated using the exponential of the fixed effects from the mixed model.
^b^SE: Standard Error.

^c^Model: reference levels: angle = Side, distance = 090 cm.

^d^Values with *P*  < .05 were considered statistically significant.

Table S2. Post-hoc pairwise comparisons for push-ups

| **Comparison** | **Estimate** | **SE**^b^ | **z.ratio** | ***P* value** |
| --- | --- | --- | --- | --- |
| Diagonal-090cm vs Front-090cm | 3.408 | 0.958 | 3.557 | .019 |
| Diagonal-090cm vs Front-180cm | 3.673 | 1.027 | 3.578 | .018 |
| Diagonal-090cm vs Front-200cm | 3.520 | 1.022 | 3.445 | .028 |
| Diagonal-090cm vs Front-360cm | 4.398 | 1.034 | 4.252 | .001 |
| Front-090cm vs Diagonal-180cm | -3.372 | 0.964 | -3.498 | .024 |
| Front-090cm vs Diagonal-200cm | -2.974 | 0.898 | -3.312 | .044 |
| Side-180cm vs Front-360cm | 3.586 | 0.940 | 3.814 | .008 |
| Diagonal-180cm vs Front-180cm | 3.636 | 1.032 | 3.524 | .022 |
| Diagonal-180cm vs Front-200cm | 3.483 | 1.027 | 3.391 | .034 |
| Diagonal-180cm vs Front-360cm | 4.362 | 1.039 | 4.197 | .002 |
| Diagonal-200cm vs Front-360cm | 3.964 | 0.977 | 4.057 | .003 |
| Side-360cm vs Front-360cm | 3.174 | 0.932 | 3.406 | .032 |
| Front-180cm vs Diagonal-200cm | -3.239 | 0.971 | -3.334 | .041 |

^a^Values with *P* < .05 were considered statistically significant.

^b^SE: Standard Error.

Table S3. Linear mixed model for push-ups

| **Factor** | **Estimate** | **SE**^c^ | **t value** | ***P* value** |
| --- | --- | --- | --- | --- |
| (Intercept) | 0.851 | 0.374 | 2.275 | .024 |
| factor(angle)Diagonal | -0.591 | 0.471 | -1.253 | .212 |
| factor(angle)Front | 0.867 | 0.483 | 1.795 | .074 |
| factor(distance)180cm | -0.442 | 0.466 | -0.948 | .344 |
| factor(distance)200cm | 0.273 | 0.471 | 0.579 | .563 |
| factor(distance)360cm | -0.013 | 0.484 | -0.028 | .978 |
| factor(angle)Diagonal:factor(distance)180cm | 0.442 | 0.654 | 0.676 | .500 |
| factor(angle)Front:factor(distance)180cm | 0.670 | 0.694 | 0.965 | .336 |
| factor(angle)Diagonal:factor(distance)200cm | -0.306 | 0.654 | -0.468 | .641 |
| factor(angle)Front:factor(distance)200cm | -0.308 | 0.698 | -0.441 | .660 |
| factor(angle)Diagonal:factor(distance)360cm | 1.202 | 0.671 | 1.791 | .075 |
| factor(angle)Front:factor(distance)360cm | 0.951 | 0.679 | 1.402 | .163 |

^a^Values with *P* < .05 were considered statistically significant.

^b^Model: reference levels: angle = Side, distance = 090 cm.

^c^SE: Standard Error.

Table S4. Post-hoc pairwise comparisons for push-ups

| **Contrast** | **Estimate** | **SE**^c^ | **df** | **t ratio** | ***P*  value** |
| --- | --- | --- | --- | --- | --- |
| Side 090cm - Front 360cm | -1.805 | 0.478 | 202.841 | -3.778 | .010 |
| Diagonal 090cm - Front 180cm | -1.686 | 0.506 | 203.843 | -3.333 | .046 |
| Diagonal 090cm - Front 360cm | -2.395 | 0.465 | 202.074 | -5.152 | < .001 |
| Side 180cm - Front 360cm | -2.246 | 0.459 | 201.747 | -4.891 | < .001 |
| Diagonal 180cm - Front 180cm | -1.686 | 0.506 | 203.783 | -3.333 | .046 |
| Diagonal 180cm - Front 360cm | -2.395 | 0.465 | 202.031 | -5.152 | < .001 |
| Front 180cm - Diagonal 200cm | 1.719 | 0.500 | 203.42 | 3.435 | .034 |
| Diagonal 200cm - Front 360cm | -2.428 | 0.459 | 201.747 | -5.287 | < .001 |
| Side 360cm - Front 360cm | -1.818 | 0.478 | 202.699 | -3.807 | .010 |

^a^Values with *P* < 0.05 were considered statistically significant.

^c^SE: Standard Error.

Table S5. Generalized linear mixed model for squats

| **Factor** | **Estimate** | **OR^a^** | **SE^b^** | **z value** | ***P* value** |
| --- | --- | --- | --- | --- | --- |
| Intercept | -2.570 | 0.077 | 0.446 | -5.755 | < .001 |
| Factor(Angle)Diagonal | 3.017 | 20.433 | 0.431 | 6.996 | < .001 |
| Factor(Angle)Front | 3.136 | 23.005 | 0.446 | 7.028 | < .001 |
| Factor(Distance)180cm | 1.472 | 4.358 | 0.468 | 3.142 | .002 |
| Factor(Distance)200cm | 2.030 | 7.615 | 0.500 | 4.058 | < .001 |
| Factor(Distance)360cm | 1.116 | 3.054 | 0.453 | 2.464 | .014 |

^a^OR: Odds Ratio calculated using the exponential of the fixed effects from the mixed model

^b^SE: Standard Error.
^c^Values with *P* < .05 were considered statistically significant.
^d^Model: reference levels: angle = Side, distance = 090 cm.

Table S6. Post-hoc pairwise comparisons for squats

| **Comparison** | **Estimate** | **SE**^c^ | **z.ratio** | ***P*  value** |
| --- | --- | --- | --- | --- |
| Diagonal-180cm vs Front-090cm | 2.810 | 0.793 | 3.542 | .020 |
| Diagonal-180cm vs Front-180cm | 3.058 | 0.812 | 3.765 | .009 |
| Diagonal-180cm vs Front-200cm | -3.271 | 0.894 | -3.658 | .014 |
| Diagonal-180cm vs Front-360cm | -4.016 | 1.147 | -3.503 | .023 |
| Front-090cm vs Diagonal-180cm | -3.155 | 0.897 | -3.517 | .022 |
| Front-090cm vs Diagonal-200cm | -3.271 | 0.894 | -3.658 | .014 |
| Side-180cm vs Front-360cm | 2.907 | 0.872 | 3.332 | .041 |
| Diagonal-180cm vs Front-180cm | 3.518 | 0.911 | 3.862 | .006 |
| Diagonal-180cm vs Front-200cm | 2.651 | 0.761 | 3.482 | .025 |
| Diagonal-180cm vs Front-360cm | 3.964 | 0.977 | 4.057 | .003 |
| Diagonal-200cm vs Front-360cm | 3.174 | 0.932 | 3.406 | .032 |
| Side-360cm vs Front-360cm | 3.239 | 0.971 | -3.334 | .041 |
| Front-180cm vs Diagonal-200cm | -2.907 | 0.872 | 3.332 | .041 |

^a^Post-hoc pairwise comparisons of classification accuracy by condition (GLMM, logit scale; squat exercise).
^b^Values with *P* < .05 were considered statistically significant.

^c^SE: Standard Error.

Table S7. Linear mixed model for squats

| **Factor** | **Estimate** | **SE^c^** | **t value** | ***P* value** |
| --- | --- | --- | --- | --- |
| Intercept | 5.000 | 0.246 | 20.299 | < .001 |
| factor(angle)Diagonal | -3.682 | 0.338 | -10.881 | < .001 |
| factor(angle)Front | -4.682 | 0.338 | -13.836 | < .001 |
| factor(distance)180cm | -4.287 | 0.342 | -12.516 | < .001 |
| factor(distance)200cm | -4.364 | 0.338 | -12.896 | < .001 |
| factor(distance)360cm | -1.910 | 0.342 | -5.578 | < .001 |
| factor(angle)Diagonal:factor(distance)180cm | 3.060 | 0.482 | 6.355 | < .001 |
| factor(angle)Front:factor(distance)180cm | 4.547 | 0.484 | 9.386 | < .001 |
| factor(angle)Diagonal:factor(distance)200cm | 3.091 | 0.478 | 6.459 | < .001 |
| factor(angle)Front:factor(distance)200cm | 4.460 | 0.485 | 9.201 | < .001 |
| factor(angle)Diagonal:factor(distance)360cm | 0.683 | 0.482 | 1.419 | .157 |
| factor(angle)Front:factor(distance)360cm | 2.688 | 0.488 | 5.513 | < .001 |

^a^Values with *P* < .05 were considered statistically significant.

^b^Model: reference levels: angle = Side, distance = 090 cm.

^c^SE: Standard Error.

^d^MAE: Mean Absolute Error.

Table S8. Post-hoc pairwise comparisons for squats

| **Contrast** | **Estimate** | **SE**^c^ | **df** | **t ratio** | ***P*  value** |
| --- | --- | --- | --- | --- | --- |
| Side 090cm - Diagonal 090cm | 3.682 | 0.338 | 224 | 10.881 | < .001 |
| Side 090cm - Front 090cm | 4.682 | 0.338 | 224 | 13.836 | < .001 |
| Side 090cm - Side 180cm | 4.287 | 0.343 | 225 | 12.514 | < .001 |
| Side 090cm - Diagonal 180cm | 4.909 | 0.338 | 224 | 14.508 | < .001 |
| Side 090cm - Front 180cm | 4.422 | 0.343 | 225 | 12.907 | < .001 |
| Side 090cm - Side 200cm | 4.364 | 0.338 | 224 | 12.896 | < .001 |
| Side 090cm - Diagonal 200cm | 4.955 | 0.338 | 224 | 14.642 | < .001 |
| Side 090cm - Front 200cm | 4.586 | 0.347 | 225 | 13.21 | < .001 |
| Side 090cm - Side 360cm | 1.910 | 0.343 | 225 | 5.577 | < .001 |
| Side 090cm - Diagonal 360cm | 4.909 | 0.338 | 224 | 14.508 | < .001 |
| Side 090cm - Front 360cm | 3.904 | 0.347 | 225 | 11.247 | < .001 |
| Diagonal 090cm - Diagonal 180cm | 1.227 | 0.338 | 224 | 3.627 | .018 |
| Diagonal 090cm - Diagonal 200cm | 1.273 | 0.338 | 224 | 3.77 | .011 |
| Diagonal 090cm - Side 360cm | -1.771 | 0.343 | 225 | -5.154 | < .001 |
| Diagonal 090cm - Diagonal 360cm | 1.227 | 0.338 | 224 | 3.627 | .018 |
| Front 090cm - Side 360cm | -2.771 | 0.343 | 225 | -8.071 | < .001 |
| Side 180cm - Side 360cm | -2.376 | 0.343 | 225 | -6.932 | < .001 |
| Diagonal 180cm - Side 360cm | -2.999 | 0.343 | 225 | -8.737 | < .001 |
| Front 180cm - Side 360cm | -2.375 | 0.347 | 225 | -6.855 | < .001 |
| Side 200cm - Side 360cm | -2.454 | 0.338 | 224 | -7.257 | < .001 |
| Diagonal 200cm - Side 360cm | -3.045 | 0.338 | 224 | -9.001 | < .001 |
| Front 200cm - Side 360cm | -2.675 | 0.347 | 225 | -7.712 | < .001 |
| Side 360cm - Diagonal 360cm | 2.999 | 0.343 | 225 | 8.737 | < .001 |
| Side 360cm - Front 360cm | 1.993 | 0.347 | 225 | 5.743 | < .001 |

^a^Values with *P* < .05 were considered statistically significant.

^c^SE: Standard Error.
